# Supplementary material for: COVID-19-Related Factors Associated with Sleep Disturbance and Suicidal Thoughts among the Taiwanese Public: A Facebook Survey
Source: Int J Environ Res Public Health. 2020 Jun 22;17(12):4479. doi: 10.3390/ijerph17124479 (PMC7345275; doi:10.3390/ijerph17124479)
Supplement: Supplementary file 1 [file ijerph-17-04479-s001.pdf]

International Journal of Environmental Research and Public Health

Table S1: Sleep disturbance and suicidal thoughts

The sleep disturbance of participants was assessed with the question: "In the past week, did you have sleep problems?"
Suicidal thoughts were evaluated by the question: "In the past week, did you ever have suicidal thoughts?"

Table S2. Worry about COVID-19

The first question was "If you were to develop flu-like symptoms tomorrow, would you be…" with the response ranging from 0 (not at all worried) to 6 (extremely worried).
The second question was "In the past one week, have you ever worried about catching COVID-19?" with the response ranging from 1 (no, never think about it) to 5 (worried about it all the time).
The third question was "Please rate the current level of your worry towards COVID-19"; the score of this item ranged from 1 (very mild) to 10 (very severe).
The fourth question was "How likely do you think it is that you will contract COVID-19?" with the response ranging from 0 (never) to 6 (certain).
The fifth question was "What do you think are your chances of getting COVID-19 over the next 1 month compared to others outside your family?" with the response ranging from 1 (not at all) to 7 (certain).

Table S3. Impact of the COVID-19 pandemic on participants' daily lives.

**These questions were as following:**
In the past week, did you<br>1) avoid going to crowded places<br>2) keep good indoor ventilation<br>3) clean or disinfect the house more often<br>4) wash your hands more often<br>5) wear a mask more often<br>6) search for information on COVID-19<br>7) miss reservations at clinics?

Table S4. Impact of the COVID-19 pandemic on social interaction.

**The questions included:**
In the past week, did you reduce the frequency of your social interaction with your<br>1) neighborhood,<br>2) family,<br>3) friends, and<br>4) colleagues or classmates?

Table S5. Academic/occupational interference by the COVID-19 pandemic.

**The question was as following:**
In the past week, did the COVID-19 pandemic interfere with your academic or occupational performance?

Table S6. Perceived social support and COVID-19-specified support.

**For social support, the questions were as following:**
In the past week were you satisfied with the support you received from your<br>1) family members<br>2) friends<br>3) colleagues or classmates?
**For COVID-19 specific support, the questions were as following:**
During the COVID-19 pandemic, did you get enough resources or support with regard to the following:<br>1) basic equipment for daily lives, such as masks or hand sanitizer<br>2) information about infection control<br>3) financial support<br>4) medical resources<br>5) mental support?

Table S7. Self-reported physical health.

**The two questions were as following:**
1. How is your physical health state compared with other people in the past week?
2. How was your physical health state compared with other people before the COVID-19 pandemic?
